# Impact of swab removal in the detection of SARS-CoV-2 weakly-positive specimens

**DOI:** 10.1099/acmi.0.000718.v3

**Published:** 2023-12-12

**Authors:** Azul Zorzoli, Susan Bennett-Slater, Alasdair MacLean, Gina McAllister, Rory Gunson, Kate Templeton

**Affiliations:** ^1^​ Department of Clinical Microbiology, NHS Lothian, Edinburgh, UK; ^2^​ West of Scotland Specialist Virology Centre, Glasgow Royal Infirmary, Glasgow, UK

**Keywords:** swabbing, SARS-CoV-2 detection, viral load, cycle threshold (Ct)

## Abstract

Removing the swab after collection can speed up diagnosis and improve the quality of laboratory procedures. This study investigates the impact of swab removal on SARS-CoV-2 detection in clinical specimens with a focus on high Cycle threshold (Ct) samples (Cts≥32). The method assessed pairs of SARS-CoV-2 samples mimicking combined throat and nose swabs and tested them on two real-time-PCR platforms; the Applied Biosystems 7500 and the Abbott Alinity. Swab removal did not significantly affect detection rates of SARS-CoV-2 samples with Ct values<32, regardless of the PCR platform. However, reduced reproducibility was seen at the endpoint limit of detection of the platforms, which meant that fewer samples with Ct values≥32 were detected in the swab removal group.

## Graphical Abstract

Early removal of swabs can accelerate laboratory work and enhance quality by decreasing contamination and errors in downstream procedures. This article aims to compare the traditional testing workflow in a diagnostic laboratory (red), with manual swab removal and aliquoting vs a swab-out laboratory workflow (blue).
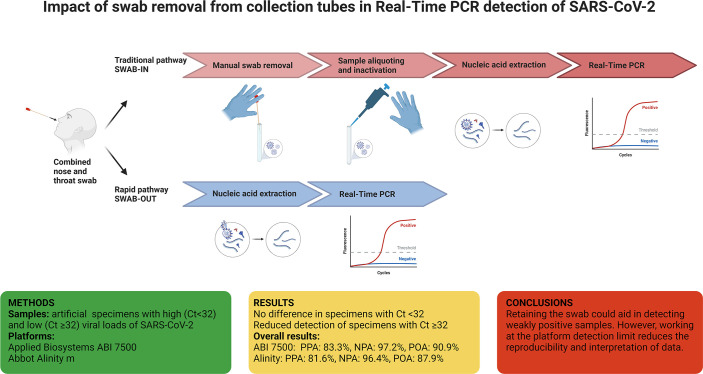

## Data Summary

A supplementary file is available with the online version of this article. All the supporting data discussed in this article can be found at https://github.com/azulzorzoli/swab_article_repository.

**Impact Statement**

Most automated Real-Time PCR testing platforms require swabs to be removed from the tubes before analysis, a time-consuming task performed by laboratory staff. The COVID-19 pandemic encouraged diagnostic laboratories to explore ways to reduce contamination and errors while improving the speed of testing. Among the alternatives, laboratories considered removing the swab immediately after specimen collection to simplify the patient-to-result path, but the impact on performance has received limited attention in the literature. This article investigates the effect of removing the swab before specimen transport and addresses the critical issue of identifying samples with low viral load. Particular attention is given to the ability of different swab-collection methods to detect SARS-CoV-2 specimens with Cycle threshold (Ct) values ≥32.

By comparing ‘swab-in’ and ‘swab-out’ methods on identical samples, this work provides valuable insights into how sample collection techniques impact the sensitivity of viral detection. The research also includes a platform performance comparison, making it relevant for laboratory practice.

The study suggests that retaining the swab after sample collection may enhance the detection rate for weakly positive samples (Ct ≥32). Although the results are preliminary and would benefit from a larger dataset, they contribute to the ongoing discussions in diagnostic laboratories about how to interpret the results from specimens with low viral load.

## Introduction

Correctly identifying positive samples, particularly in healthcare settings, is crucial in guiding public health measures and mitigating the spread of viral diseases [1, 2]. Specimens with low viral loads show high Cycle threshold (Ct) results in real-time PCR tests [3, 4]. These weak positives can be seen in patients shedding residual nucleic acid from a previous infection, but also because of poor swab collection technique. High Ct samples may also occur in pre-symptomatic or asymptomatic individuals.

Greene [5] and Stevenson [6] discussed sample collection strategies, mainly using swab removal as a potential pre-analytical strategy. Early removal of swabs can accelerate laboratory work and enhance quality by decreasing contamination and errors in downstream procedures. However, little attention has been given to the potential effect of swab-collection methods in detecting specimens with low viral load. This work compares swab-in and swab-out methods on identical samples in parallel, focusing on specimens with Ct values≥32. A platform performance comparison was also included in this study using the Applied Biosystems 7500 and the Abbott Alinity.

## Methods and results

Thirty-one positive and twenty-seven negative SARS-CoV-2 testing samples mimicking combined throat and nose swabs were generated from serial dilutions of clinical specimens in Viral PCR Sample Solution (VPSS), the transport inactivation media routinely used in the laboratory. For the ‘swab-in’ samples, the swab was swirled in VPSS for 5 s, the shaft was broken, and the tube closed. For the ‘swab-out’ specimens, the swab was submerged into VPSS, swirled five times, squeezed against the tube’s wall and then discarded. Samples were incubated overnight at room temperature to simulate collection-to-reception time. The sample sets were tested through A) a partially automated workflow using an Easymag for nucleic acid extraction followed by the Applied Biosystems 7500 and the Altona RealStar SARS-CoV-2 RT-PCR Kit 1.0, or B) the fully automated platform Alinity *m* from Abbot.

Table 1 shows the average Cycle threshold (Ct) or Copy Number (CN) values measured for each sample group for each platform. Of note, Ct and CN values are not directly comparable across different platforms. CN numbers are often lower than Ct values, with the first estimating the number of gene or genomic region copies and the second counting the cycles needed to amplify a target gene to a detectable level. Table S1, available in the online version of this article contains all platforms and methods’ individual Ct/CN values.

**Table 1.** Summary statistics for each platform and category

Platform	Method	Ct/CN <32 Group	Ct/CN ≥32 Group
ABI (E gene)	Swab-in	Average Ct=29.31	Average Ct=36.52
		Range: 25.27–31.92	Range: 32.14–38.25
	Swab-out	Average Ct=28.84	Average Ct=36.35
		Range: 25.42–31.15	Range: 32.08–39.49
Alinity m	Swab-in	Average CN=28.05	Average CN=35.67
		Range: 21.86–31.85	Range: 32.12–38.80
	Swab-out	Average CN=27.84	Average CN=35.56
		Range: 21.93–31.00	Range: 32.03–40.83

**Fig. 1.**
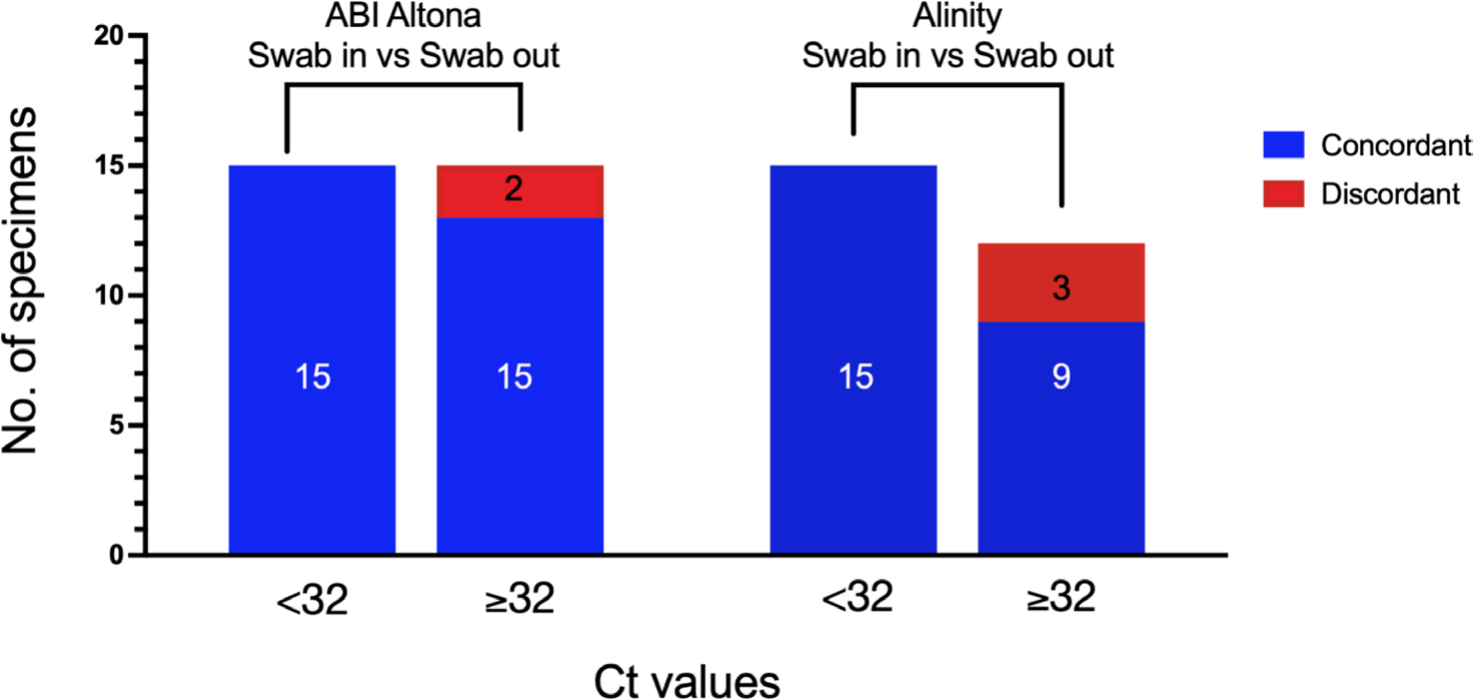
Agreement between swab-in and swab-out methods. Comparison of concordant (true positives, in blue) and discordant (false negatives, in red) results observed after swab removal for the <32 and ≥32 groups for the ABI (left) and Alinity (right) Real-Time PCR Platforms. Inside the bar charts are the number of samples in each group.

Fig. 1 summarises the agreement between the two swab methodologies for each platform and Ct/CN group. The results show no discordant results for samples with strong or moderately strong viral loads (Ct <32), but several high-Ct (≥32) samples were not detected. The expected variation in sample collection resulted in some samples failing detection using the swab-in method but being positive after removing the swab. These inconsistencies were interpreted cautiously, with only the positive samples in both methods or positive in swab-in and negative in swab-out, being considered for analysis. Our data analysis strategy considered samples positive only when both replicates were detected. Despite this, a paired t-test revealed no statistical differences between the means of the swab-in vs swab-out samples regardless of the swab group tested (strongly positive and moderately positive, with Ct <32, and weakly positive with Ct ≥32) and the platform used (Figs S1 and S2, Tables S2 and S4).

The datasets were analysed for each platform individually, comparing the expected results (swab-in) to the ones resulting from the swab removal. Table 2 details the Positive Percentage Agreement (PPA), Negative Percentage Agreement (NPA) and Percentage Overall Agreement (POA) results when comparing the swab-in vs swab-out method. ABI performed slightly better than Alinity when comparing the overall results (POA 90.9 % vs 87.9 %). When testing samples with Ct <32, these two platforms showed no detrimental effect, with PNA, NPA and POA of 100.00 %. However, lower PPA and PNA were seen for the Ct ≥32 groups, with ABI performing better than the Alinity platform (PPA 77.3 % vs 65.0 %). The contingency tables in the supplementary materials describe these results in more detail (Table S3).

**Table 2.** Comparison of swab-in vs swab-out methods for the detection of SARS-CoV-2 (Ct ≥32) using the Applied Biosystems 7500 Fast Real-Time PCR platform. Summary results for the comparison between the swab-in vs swab-out methods

Group	Platform	PPA	NPA	POA	no. of positive samples tested	no. of negative samples tested
All samples	Alinity	81.6 % (CI 95 %=66.6–90.8 %)	96.4 % (CI 95 %=82.3–99.4 %)	87.9 % (CI 95 %=77.9–93.7 %)	38	28
CN <32	Alinity	100.0 % (CI 95 %=82.4–100.0 %)	100.0 % (CI 95 %=87.5–100.0 %)	100.0 % (CI 95 %=92.1–100.0 %)	18	27
CN ≥32	Alinity	65.0 % (CI 95 %=43.3–91.9 %)	96.4 % (CI 95 %=82.3–99.4 %)	83.3 % (CI 95 %=70.9–91.3 %)	20	28
All samples	ABI	83.3 % (CI 95 %=66.4–92.7 %)	97.2 % (CI 95 %=85.8–99.5 %)	90.9 % (CI 95 %=81.6–95.8 %)	30	36
Ct <32	ABI	100.0 % (CI 95 %=67.6–100.0 %)	100.0 % (CI 95 %=90.1–100.0 %)	100.0 % (CI 95 %=91.8–100.0 %)	8	35
Ct ≥32	ABI	77.3 % (CI 95 %=56.6–89.9 %)	97.2 % (CI 95 %=85.8–99.5 %)	89.7 % (CI 95 %=79.2–95.2 %)	22	36

The COVID-19 pandemic saw the development of new tests for detecting SARS-CoV-2 and a plethora of information describing assays, platforms, and sample collection strategies [7, 8]. In this work, we evaluated if discarding the swab after sample collection would increase the risk detection rate, specifically for weakly positive samples (Ct ≥32). We saw no impact in samples with strongly positive and moderately positive viral loads (here defined as samples with Cts<32) but we recorded a lower detection rate after swab removal in samples with Ct values above 32.

A careful methodology was used to collect paired specimen samples to reduce inconsistencies, but methodological differences may have still impacted the results presented here. Issues with this collection methodology can introduce errors that confound the interpretation of clinical results. For instance, low viral load samples might result from poorly collected swab specimens. However, they might also reflect the initial stages of an infectious process or the residual viral RNA shedding from individuals who are no longer infectious [9, 10]. Although this article does not aid in clinical interpretation, it can be used to make reporting decisions for samples with low viral loads.

Many laboratories have long-standing protocols for rejecting empty containers and only processing those with a retained swab to minimise processing errors. Those pathways could be revised in light of these results, as specimens with Ct <32 are unaffected by the swab removal. By doing so, laboratories can ensure the processing of more specimens and provide high-quality results to their patients and healthcare providers.

Our data suggest that retaining the swab could help detect weakly positive samples. However, a larger data set would be required to reach a definitive conclusion. It is possible that the reduction in the assay’s ability to consistently detect low viral loads, which occurs when working at the platform’s detection levels, compounded by the expected variations in the swirling technique, is leading to the observed decrease in the detection rate.

## Supplementary Data

Supplementary material 1
