# Supplementary material for: Impact of swab removal in the detection of SARS-CoV-2 weakly-positive specimens
Source: Access Microbiol. 2023 Dec 12;5(12):000718.v3. doi: 10.1099/acmi.0.000718.v3 (PMC10765045; doi:10.1099/acmi.0.000718.v3)
Supplement: Supplementary material 1 [file acmi-5-718.v3-s001.pdf]

# Annex Swab Paper

*Table S1*: Paired Ct and CN values for samples (S) and controls (C) in the ABI and Alinity platforms

| sample_id | swab_status | Ct_average | result | platform |
|---|---|---|---|---|
| S10D1 | Swab_in | 32.2745 | positive | ABI |
| S10D1 | Swab_out | 32.66615 | positive | ABI |
| S10D2 | Swab_in | 35.6901 | positive | ABI |
| S10D2 | Swab_out | 36.36565 | positive | ABI |
| S10D3 | Swab_in | 38.56985 | positive | ABI |
| S10D3 | Swab_out | | negative | ABI |
| S10D4 | Swab_in | | negative | ABI |
| S10D4 | Swab_out | | negative | ABI |
| S10D5 | Swab_in | | negative | ABI |
| S10D5 | Swab_out | | negative | ABI |
| S10D6 | Swab_in | | negative | ABI |
| S10D6 | Swab_out | | negative | ABI |
| S1D1 | Swab_in | 28.75755 | positive | ABI |
| S1D1 | Swab_out | 29.81525 | positive | ABI |
| S1D2 | Swab_in | 32.5157 | positive | ABI |
| S1D2 | Swab_out | 33.4679 | positive | ABI |
| S1D3 | Swab_in | 35.4992 | positive | ABI |
| S1D3 | Swab_out | 36.8991 | positive | ABI |
| S1D4 | Swab_in | | negative | ABI |
| S1D4 | Swab_out | | negative | ABI |
| S1D5 | Swab_in | | negative | ABI |
| S1D5 | Swab_out | | negative | ABI |
| S1D6 | Swab_in | | negative | ABI |
| S1D6 | Swab_out | | negative | ABI |
| S2D1 | Swab_in | 29.428 | positive | ABI |
| S2D1 | Swab_out | 30.01295 | positive | ABI |
| S2D2 | Swab_in | 33.14045 | positive | ABI |
| S2D2 | Swab_out | 35.45635 | positive | ABI |
| S2D3 | Swab_in | 36.5602 | positive | ABI |
| S2D3 | Swab_out | 37.02375 | positive | ABI |
| S2D4 | Swab_in | 37.5758 | positive | ABI |
| S2D4 | Swab_out | | negative | ABI |

| sample_id | swab_status | CN_average | result | platform |
|---|---|---|---|---|
| S10D1 | Swab_in | 29.72 | positive | Alinity |
| S10D1 | Swab_out | 33.06 | positive | Alinity |
| S10D2 | Swab_in | 33.635 | positive | Alinity |
| S10D2 | Swab_out | | negative | Alinity |
| S10D3 | Swab_in | 36.815 | positive | Alinity |
| S10D3 | Swab_out | 36.93 | positive | Alinity |
| S10D4 | Swab_in | | negative | Alinity |
| S10D4 | Swab_out | | negative | Alinity |
| S10D5 | Swab_in | | negative | Alinity |
| S10D5 | Swab_out | | negative | Alinity |
| S10D6 | Swab_in | | negative | Alinity |
| S10D6 | Swab_out | | negative | Alinity |
| S1D1 | Swab_in | 26.45 | positive | Alinity |
| S1D1 | Swab_out | 27.205 | positive | Alinity |
| S1D2 | Swab_in | 29.45 | positive | Alinity |
| S1D2 | Swab_out | 30.715 | positive | Alinity |
| S1D3 | Swab_in | 33.4 | positive | Alinity |
| S1D3 | Swab_out | 33.75 | positive | Alinity |
| S1D4 | Swab_in | 36.78 | positive | Alinity |
| S1D4 | Swab_out | 38.38 | positive | Alinity |
| S1D5 | Swab_in | | negative | Alinity |
| S1D5 | Swab_out | | negative | Alinity |
| S1D6 | Swab_in | | negative | Alinity |
| S1D6 | Swab_out | | negative | Alinity |
| S2D1 | Swab_in | 25.99 | positive | Alinity |
| S2D1 | Swab_out | 26.56 | positive | Alinity |
| S2D2 | Swab_in | 29.695 | positive | Alinity |
| S2D2 | Swab_out | 29.985 | positive | Alinity |
| S2D3 | Swab_in | 32.935 | positive | Alinity |
| S2D3 | Swab_out | 33.515 | positive | Alinity |
| S2D4 | Swab_in | 37.565 | positive | Alinity |
| S2D4 | Swab_out | 37.585 | positive | Alinity |

| | | | | |
|---|---|---|---|---|
| S2D5 | Swab_in | | negative | ABI |
| S2D5 | Swab_out | | negative | ABI |
| S2D6 | Swab_in | | negative | ABI |
| S2D6 | Swab_out | | negative | ABI |
| S3D1 | Swab_in | 32.07605 | positive | ABI |
| S3D1 | Swab_out | 32.1414 | positive | ABI |
| S3D2 | Swab_out | 35.18175 | positive | ABI |
| S3D2 | Swab_in | 36.5025 | positive | ABI |
| S3D3 | Swab_out | 36.4844 | positive | ABI |
| S3D3 | Swab_in | | negative | ABI |
| S3D4 | Swab_in | | negative | ABI |
| S3D4 | Swab_out | | negative | ABI |
| S3D5 | Swab_in | | negative | ABI |
| S3D5 | Swab_out | | negative | ABI |
| S3D6 | Swab_in | | negative | ABI |
| S3D6 | Swab_out | | negative | ABI |
| S4D1 | Swab_in | 28.4386 | positive | ABI |
| S4D1 | Swab_out | 28.72945 | positive | ABI |
| S4D2 | Swab_out | 32.2191 | positive | ABI |
| S4D2 | Swab_in | 32.6 | positive | ABI |
| S4D3 | Swab_in | 35.4802 | positive | ABI |
| S4D3 | Swab_out | 36.10315 | positive | ABI |
| S4D4 | Swab_in | 38.1931 | positive | ABI |
| S4D4 | Swab_out | | negative | ABI |
| S4D5 | Swab_in | | negative | ABI |
| S4D5 | Swab_out | | negative | ABI |
| S4D6 | Swab_in | | negative | ABI |
| S4D6 | Swab_out | | negative | ABI |
| S5D1 | Swab_out | 25.27315 | positive | ABI |
| S5D1 | Swab_in | 25.41595 | positive | ABI |
| S5D2 | Swab_out | 28.8412 | positive | ABI |
| S5D2 | Swab_in | 28.91475 | positive | ABI |
| S5D3 | Swab_out | 31.9235 | positive | ABI |
| S5D3 | Swab_in | 32.8178 | positive | ABI |
| S5D4 | Swab_in | 36.23615 | positive | ABI |
| S5D4 | Swab_out | 36.51545 | positive | ABI |
| S5D5 | Swab_in | 39.4948 | positive | ABI |
| S5D5 | Swab_out | | negative | ABI |
| S5D6 | Swab_in | | negative | ABI |

| | | | | |
|---|---|---|---|---|
| S2D5 | Swab_in | 38.72 | positive | Alinity |
| S2D5 | Swab_out | | negative | Alinity |
| S2D6 | Swab_in | | negative | Alinity |
| S2D6 | Swab_out | | negative | Alinity |
| S3D1 | Swab_in | 27.165 | positive | Alinity |
| S3D1 | Swab_out | 29.59 | positive | Alinity |
| S3D2 | Swab_in | 30.57 | positive | Alinity |
| S3D2 | Swab_out | 32.705 | positive | Alinity |
| S3D3 | Swab_in | 35.105 | positive | Alinity |
| S3D3 | Swab_out | 36.575 | positive | Alinity |
| S3D4 | Swab_in | 38.805 | positive | Alinity |
| S3D4 | Swab_out | | negative | Alinity |
| S3D5 | Swab_in | | negative | Alinity |
| S3D5 | Swab_out | | negative | Alinity |
| S3D6 | Swab_in | | negative | Alinity |
| S3D6 | Swab_out | | negative | Alinity |
| S4D1 | Swab_in | 23.925 | positive | Alinity |
| S4D1 | Swab_out | 25.47 | positive | Alinity |
| S4D2 | Swab_in | 27.48 | positive | Alinity |
| S4D2 | Swab_out | 29.355 | positive | Alinity |
| S4D3 | Swab_in | 30.73 | positive | Alinity |
| S4D3 | Swab_out | 32.035 | positive | Alinity |
| S4D4 | Swab_in | 34.06 | positive | Alinity |
| S4D4 | Swab_out | 36.27 | positive | Alinity |
| S4D5 | Swab_in | 36.445 | positive | Alinity |
| S4D5 | Swab_out | 38.765 | positive | Alinity |
| S4D6 | Swab_in | | negative | Alinity |
| S4D6 | Swab_out | | negative | Alinity |
| S5D1 | Swab_in | 21.855 | positive | Alinity |
| S5D1 | Swab_out | 21.935 | positive | Alinity |
| S5D2 | Swab_out | 25.375 | positive | Alinity |
| S5D2 | Swab_in | 25.76 | positive | Alinity |
| S5D3 | Swab_out | 28.95 | positive | Alinity |
| S5D3 | Swab_in | 29.47 | positive | Alinity |
| S5D4 | Swab_in | 32.395 | positive | Alinity |
| S5D4 | Swab_out | 32.71 | positive | Alinity |
| S5D5 | Swab_in | 35.585 | positive | Alinity |
| S5D5 | Swab_out | 35.915 | positive | Alinity |
| S5D6 | Swab_out | 39.42 | positive | Alinity |

| | | | | |
|---|---|---|---|---|
| S5D6 | Swab_out | | negative | ABI |
| S6D1 | Swab_out | 30.45425 | positive | ABI |
| S6D1 | Swab_in | 31.14705 | positive | ABI |
| S6D2 | Swab_out | 35.22445 | positive | ABI |
| S6D2 | Swab_in | 35.50715 | positive | ABI |
| S6D3 | Swab_out | 37.7775 | positive | ABI |
| S6D3 | Swab_in | 39.57 | positive | ABI |
| S6D4 | Swab_in | | negative | ABI |
| S6D4 | Swab_out | | negative | ABI |
| S6D5 | Swab_in | | negative | ABI |
| S6D5 | Swab_out | | negative | ABI |
| S6D6 | Swab_in | | negative | ABI |
| S6D6 | Swab_out | | negative | ABI |
| S8D1 | Swab_in | 35.82885 | positive | ABI |
| S8D1 | Swab_out | 36.174 | positive | ABI |
| S8D2 | Swab_in | 35.0119 | positive | ABI |
| S8D2 | Swab_out | | negative | ABI |
| S8D3 | Swab_in | | negative | ABI |
| S8D3 | Swab_out | | negative | ABI |
| S8D4 | Swab_in | | negative | ABI |
| S8D4 | Swab_out | | negative | ABI |
| S8D5 | Swab_in | | negative | ABI |
| S8D5 | Swab_out | | negative | ABI |
| S8D6 | Swab_in | | negative | ABI |
| S8D6 | Swab_out | | negative | ABI |
| S9D1 | Swab_in | 27.1921 | positive | ABI |
| S9D1 | Swab_out | 28.04645 | positive | ABI |
| S9D2 | Swab_in | 30.60165 | positive | ABI |
| S9D2 | Swab_out | 31.8407 | positive | ABI |
| S9D3 | Swab_in | 34.3881 | positive | ABI |
| S9D3 | Swab_out | 35.03785 | positive | ABI |
| S9D4 | Swab_in | 37.4859 | positive | ABI |
| S9D4 | Swab_out | 38.25325 | positive | ABI |
| S9D5 | Swab_in | | negative | ABI |
| S9D5 | Swab_out | | negative | ABI |
| S9D6 | Swab_in | | negative | ABI |
| S9D6 | Swab_out | | negative | ABI |
| C1D1 | Swab_in | | negative | ABI |
| C1D1 | Swab_out | | negative | ABI |

| | | | | |
|---|---|---|---|---|
| S5D6 | Swab_in | | negative | Alinity |
| S6D1 | Swab_out | 27.295 | positive | Alinity |
| S6D1 | Swab_in | 27.725 | positive | Alinity |
| S6D2 | Swab_out | 31.005 | positive | Alinity |
| S6D2 | Swab_in | 31.345 | positive | Alinity |
| S6D3 | Swab_out | 33.94 | positive | Alinity |
| S6D3 | Swab_in | 34.67 | positive | Alinity |
| S6D4 | Swab_in | 38.285 | positive | Alinity |
| S6D4 | Swab_out | | negative | Alinity |
| S6D5 | Swab_in | | negative | Alinity |
| S6D5 | Swab_out | | negative | Alinity |
| S6D6 | Swab_in | | negative | Alinity |
| S6D6 | Swab_out | | negative | Alinity |
| S8D1 | Swab_in | 32.125 | positive | Alinity |
| S8D1 | Swab_out | 32.58 | positive | Alinity |
| S8D2 | Swab_in | 35.3 | positive | Alinity |
| S8D2 | Swab_out | | negative | Alinity |
| S8D3 | Swab_in | 39.63 | positive | Alinity |
| S8D3 | Swab_out | | negative | Alinity |
| S8D4 | Swab_in | | negative | Alinity |
| S8D4 | Swab_out | | negative | Alinity |
| S8D5 | Swab_in | | negative | Alinity |
| S8D5 | Swab_out | | negative | Alinity |
| S8D6 | Swab_in | | negative | Alinity |
| S8D6 | Swab_out | | negative | Alinity |
| S9D1 | Swab_in | 24.29 | positive | Alinity |
| S9D1 | Swab_out | 26.49 | positive | Alinity |
| S9D2 | Swab_in | 28.755 | positive | Alinity |
| S9D2 | Swab_out | 29.87 | positive | Alinity |
| S9D3 | Swab_in | 31.85 | positive | Alinity |
| S9D3 | Swab_out | 35.045 | positive | Alinity |
| S9D4 | Swab_in | 35.415 | positive | Alinity |
| S9D4 | Swab_out | 40.83 | positive | Alinity |
| S9D5 | Swab_in | 38.075 | positive | Alinity |
| S9D5 | Swab_out | | negative | Alinity |
| S9D6 | Swab_in | | negative | Alinity |
| S9D6 | Swab_out | | negative | Alinity |
| C1D1 | Swab_in | | negative | Alinity |
| C1D1 | Swab_out | | negative | Alinity |

| | | | | |
|---|---|---|---|---|
| C1D2 | Swab_in | | negative | ABI |
| C1D2 | Swab_out | | negative | ABI |
| C1D3 | Swab_in | | negative | ABI |
| C1D3 | Swab_out | | negative | ABI |
| C1D4 | Swab_in | | negative | ABI |
| C1D4 | Swab_out | | negative | ABI |
| C1D5 | Swab_in | | negative | ABI |
| C1D5 | Swab_out | | negative | ABI |
| C1D6 | Swab_in | | negative | ABI |
| C1D6 | Swab_out | | negative | ABI |
| C2D1 | Swab_in | | negative | ABI |
| C2D1 | Swab_out | | negative | ABI |
| C2D2 | Swab_in | | negative | ABI |
| C2D2 | Swab_out | | negative | ABI |
| C2D3 | Swab_in | | negative | ABI |
| C2D3 | Swab_out | | negative | ABI |
| C2D4 | Swab_in | | negative | ABI |
| C2D4 | Swab_out | | negative | ABI |
| C2D5 | Swab_in | | negative | ABI |
| C2D5 | Swab_out | | negative | ABI |
| C2D6 | Swab_in | | negative | ABI |
| C2D6 | Swab_out | | negative | ABI |

| | | | | |
|---|---|---|---|---|
| C1D2 | Swab_in | | negative | Alinity |
| C1D2 | Swab_out | | negative | Alinity |
| C1D3 | Swab_in | | negative | Alinity |
| C1D3 | Swab_out | | negative | Alinity |
| C1D4 | Swab_in | | negative | Alinity |
| C1D4 | Swab_out | | negative | Alinity |
| C1D5 | Swab_in | | negative | Alinity |
| C1D5 | Swab_out | | negative | Alinity |
| C1D6 | Swab_in | | negative | Alinity |
| C1D6 | Swab_out | | negative | Alinity |
| C2D1 | Swab_in | | negative | Alinity |
| C2D1 | Swab_out | | negative | Alinity |
| C2D2 | Swab_in | | negative | Alinity |
| C2D2 | Swab_out | | negative | Alinity |
| C2D3 | Swab_in | | negative | Alinity |
| C2D3 | Swab_out | | negative | Alinity |
| C2D4 | Swab_in | | negative | Alinity |
| C2D4 | Swab_out | | negative | Alinity |
| C2D5 | Swab_in | | negative | Alinity |
| C2D5 | Swab_out | | negative | Alinity |
| C2D6 | Swab_in | | negative | Alinity |
| C2D6 | Swab_out | | negative | Alinity |

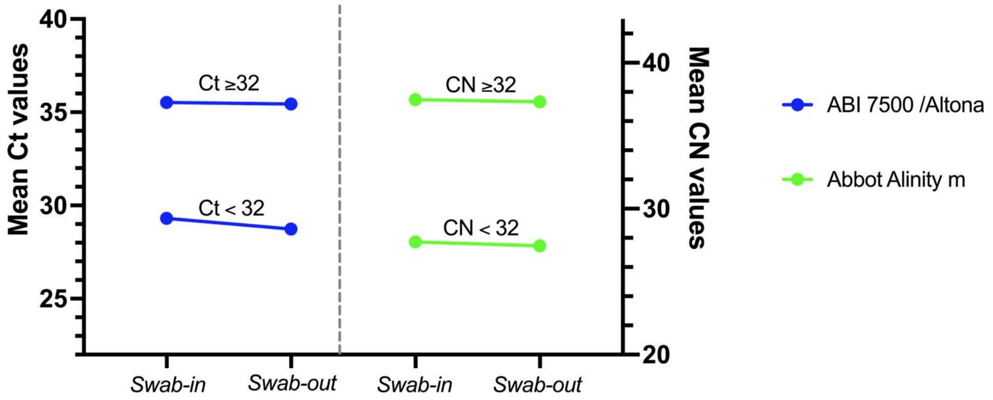


*Figure S1:* Comparison of Ct mean values before and after swab removal for the moderate-high Ct (<32) and high Ct groups (≥32).

*Table S2*: Paired t-test results comparing the mean Ct values for each group and platform

| Platform | Group | p value | CI 95% | Mean diff |
|---|---|---|---|---|
| Alinity | All | 0.8689 | 3.2129 - 3.7673 | 0.2772 |
| Alinity | CN <32 | 0.5268 | 2.8932 - 1.5541 | -0.6696 |
| Alinity | CN ≥32 | 0.9631 | -1.5493 - 1.6201 | 0.035277 |
| ABI | All | 0.3437 | -3.8388 - 1.4576 | -0.7782 |
| ABI | Ct <32 | 0.5441 | 1.5532 - 2.7003 | 0.557 |
| ABI | Ct ≥32 | 0.6893 | -1.2016 - 0.8145 | -0.0.1936 |

Table S3: Contingency tables for each group and platform

| Alinity - Overall Results | | | | |
|---|---|---|---|---|
| | | **Swab in method** | | |
| | | Positive | Negative | **Total** |
| **Swab Out method** | Positive | 31 | 1 | **32** |
| | Negative | 7 | 27 | **34** |
| | **Total** | **38** | **28** | **66** |

| Altona/ABI - Overall Results | | | | |
|---|---|---|---|---|
| | | **Swab in method** | | |
| | | Positive | Negative | **Total** |
| **Swab Out method** | Positive | 25 | 1 | **26** |
| | Negative | 5 | 35 | **40** |
| | **Total** | **30** | **36** | **66** |

| Alinity - CN <32 group | | | | |
|---|---|---|---|---|
| | | **Swab in method** | | |
| | | Positive | Negative | **Total** |
| **Swab Out method** | Positive | 18 | 0 | **18** |
| | Negative | 0 | 27 | **27** |
| | **Total** | **18** | **27** | **45** |

| Altona/ABI - Ct <32 group | | | | |
|---|---|---|---|---|
| | | **Swab in method** | | |
| | | Positive | Negative | **Total** |
| **Swab Out method** | Positive | 8 | 0 | **8** |
| | Negative | 0 | 35 | **35** |
| | **Total** | **8** | **35** | **43** |

| Alinity - CN ≥32 group | | | | |
|---|---|---|---|---|
| | | **Swab in method** | | |
| | | Positive | Negative | **Total** |
| **Swab Out method** | Positive | 13 | 1 | **14** |
| | Negative | 7 | 27 | **34** |
| | **Total** | **20** | **28** | **48** |

| Altona/ABI - Ct ≥32 group | | | | |
|---|---|---|---|---|
| | | **Swab in method** | | |
| | | Positive | Negative | **Total** |
| **Swab Out method** | Positive | 17 | 1 | **18** |
| | Negative | 5 | 35 | **40** |
| | **Total** | **22** | **36** | **58** |

Some samples were not detected in the swab-in method but were captured after removing the swab, but the results should be interpreted cautiously. Our data analysis strategy considered samples positive only when both replicates were detected. For instance, sample S5D6 was considered negative in the Alinity because only one replicate was captured in the swab-in method,

which had a very high CN value of CN 40.78. The corresponding swab-out pair samples were detected, with CNs of 40.69 and 38.15. The set was considered positive since these two samples from the swab-out method were detected.

Similarly, sample S3D3 in the SARS-Cov-2 Altona/ABI assay had a single Ct of 39.05 for the swab-in method but two values of Ct 38.54 and 38.37 after removing the swab. Boxplot figures comparing the distribution of samples and a table detailing the complete statistics can be found in the supplementary materials (**Figure S2, Table S4**).

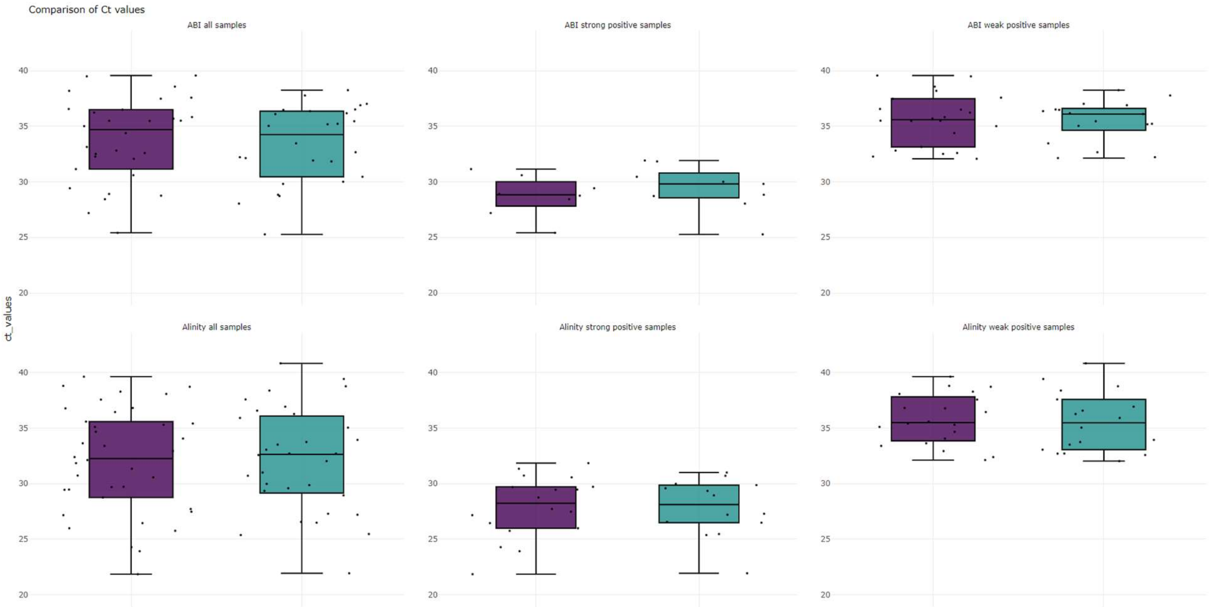


*Figure S2:* Comparison of Ct mean values before and after swab removal for the moderate-high Ct and high Ct groups.

*Table S4*: Summary statistics for all groups, conditions and platforms.

| Method | Platform | Group | Min | Max | Median | Mean | 1st Qu. | 3rd Qu. |
|---|---|---|---|---|---|---|---|---|
| Swab in | Alinity | All | 21.93 | 40.83 | 32.64 | 32.18 | 28.93 | 35.54 |
| Swab out | Alinity | All | 28.93 | 39.63 | 32.26 | 32.05 | 29.25 | 36.00 |
| Swab in | Alinity | CN <32 | 21.86 | 31.85 | 28.24 | 28.05 | 26.11 | 29.71 |
| Swab out | Alinity | CN <32 | 21.93 | 31.00 | 28.12 | 27.84 | 26.51 | 29.80 |
| Swab in | Alinity | CN ≥32 | 35.05 | 40.83 | 37.26 | 37.57 | 36.35 | 40.83 |
| Swab out | Alinity | CN ≥32 | 35.10 | 39.63 | 36.61 | 36.69 | 35.46 | 37.38 |
| Swab in | ABI | All | 25.27 | 38.25 | 34.25 | 33.38 | 31.38 | 36.44 |
| Swab out | ABI | All | 25.42 | 39.57 | 34.7 | 33.46 | 30.80 | 36.32 |
| Swab in | ABI | Ct <32 | 25.27 | 31.92 | 29.33 | 29.31 | 28.13 | 29.72 |
| Swab out | ABI | Ct <32 | 25.42 | 31.15 | 28.84 | 28.74 | 28.56 | 30.80 |
| Swab in | ABI | Ct ≥32 | 32.14 | 38.25 | 35.47 | 36.52 | 35.04 | 36.56 |
| Swab out | ABI | Ct ≥32 | 32.08 | 39.49 | 35.69 | 35.44 | 35.04 | 36.52 |
